# Rituximab-induced serum sickness in progressive transformation of germinal centers immunoglobulin G4-related disease: a case report

**DOI:** 10.1186/s13256-025-05222-1

**Published:** 2025-04-19

**Authors:** Sharanya Kumar, Rahul Tuli, Allen Seylani, Eric Abedi, Alexander Friedman

**Affiliations:** 1 Internal Medicine, Riverside University Health System (RUHS), Moreno Valley, USA; 2 School of Medicine, University of California, Riverside, Riverside, USA

**Keywords:** Rituximab, RISS, IgG4-RD, Serum sickness, PTGC, Hypersensitivity, Case report

## Abstract

**Introduction:**

Serum sickness, a type III hypersensitivity reaction, can arise from various triggers such as vaccines, anti-venoms, and certain medications, particularly monoclonal antibodies. Rituximab, a monoclonal antibody targeting CD20 on B-cells, is known to induce a subtype of this reaction called rituximab-induced serum sickness, which primarily affects those with rheumatologic diseases.

**Case presentation:**

A 34-year-old Mexican–American female patient with focal segmental glomerulosclerosis was treated with rituximab for concomitant immunoglobulin G4-related disease. After her third infusion, she developed a rash, myalgias, arthralgias, and proteinuria, leading to a diagnosis of rituximab-induced serum sickness, which resolved with a prednisone taper.

**Conclusion:**

Serum sickness should be considered as a potential differential diagnosis in patients exhibiting nonspecific systemic symptoms and dermatologic manifestations following exposure to a triggering factor, such as a newly administered monoclonal antibody medication. This is the first known case of rituximab-induced serum sickness in the context of immunoglobulin G4-related disease.

## Background

Immunoglobulin G4-related diseases (IgG4-RD) encompass a spectrum of fibroinflammatory conditions that can affect various organs including the pancreas, biliary tract, and lymph nodes. These disorders are characterized by the dense accumulation of IgG4-positive plasma cells, resulting in organ enlargement and tissue damage [1]. Rituximab, a monoclonal antibody targeting CD20 on B-cells, has emerged as a promising treatment for IgG4-RD but carries associated risks. Rituximab can trigger both acute reactions, such as allergies and anaphylaxis, as well as delayed responses such as serum sickness, particularly in patients with preexisting rheumatologic conditions [2]. The pathophysiology of serum sickness is driven by the formation of immune complexes, which develop when antigens bind to antibodies. Normally, phagocytes effectively clear these complexes, but when the clearance system is impaired, immune complexes can accumulate and precipitate within tissues, triggering an inflammatory cascade [3]. Serum sickness, a type III hypersensitivity reaction, typically manifests 1–2 weeks following rituximab exposure and is characterized by a triad of fever, rash, and polyarthralgia [4]. Here we present a case of rapid-onset rituximab-induced serum sickness (RISS) following three doses of rituximab in a patient with progressive transformation of germinal centers (PTGC) IgG4-RD.

## Case presentation

**Fig. 1:**
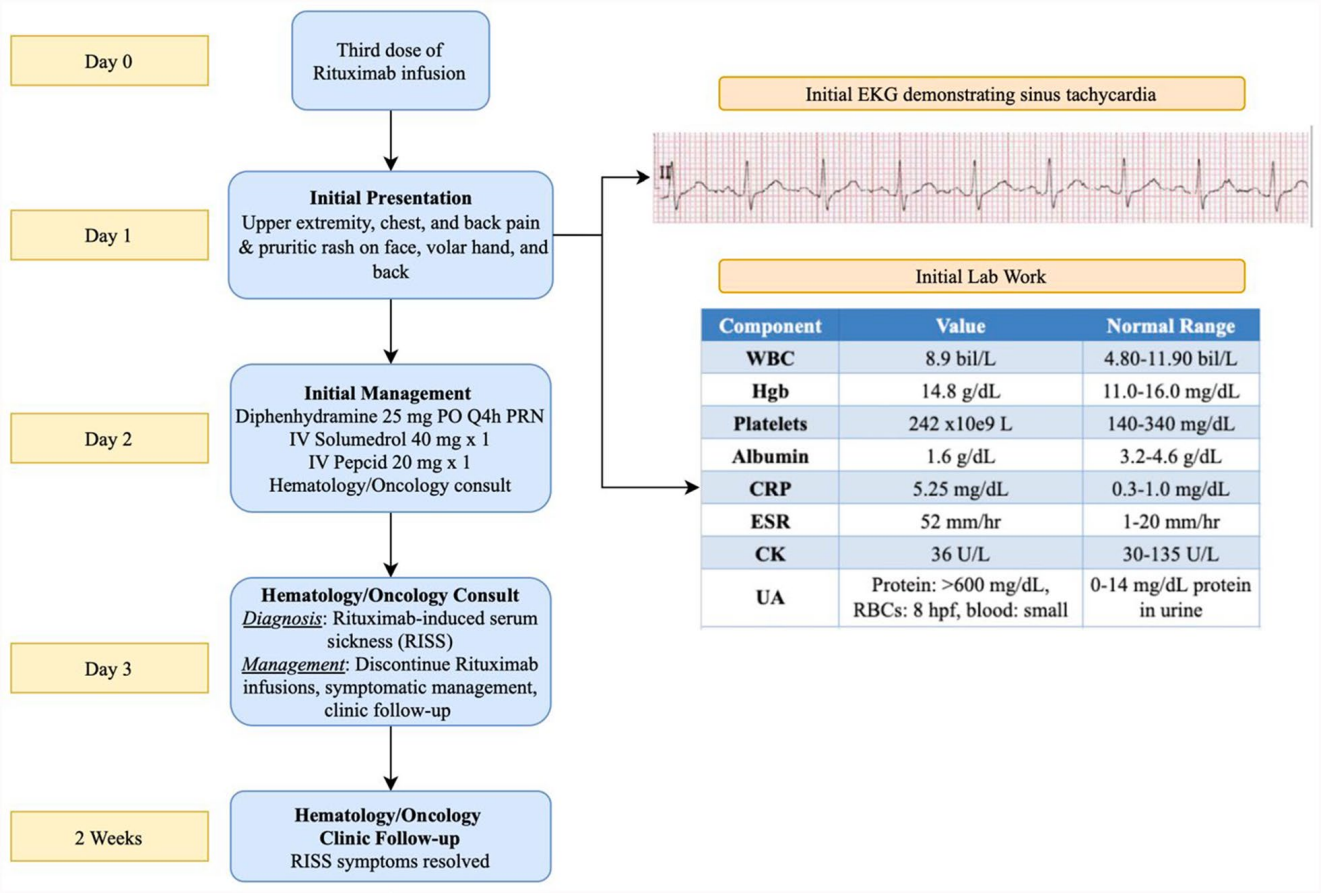
Case presentation timeline

**Fig. 2:**
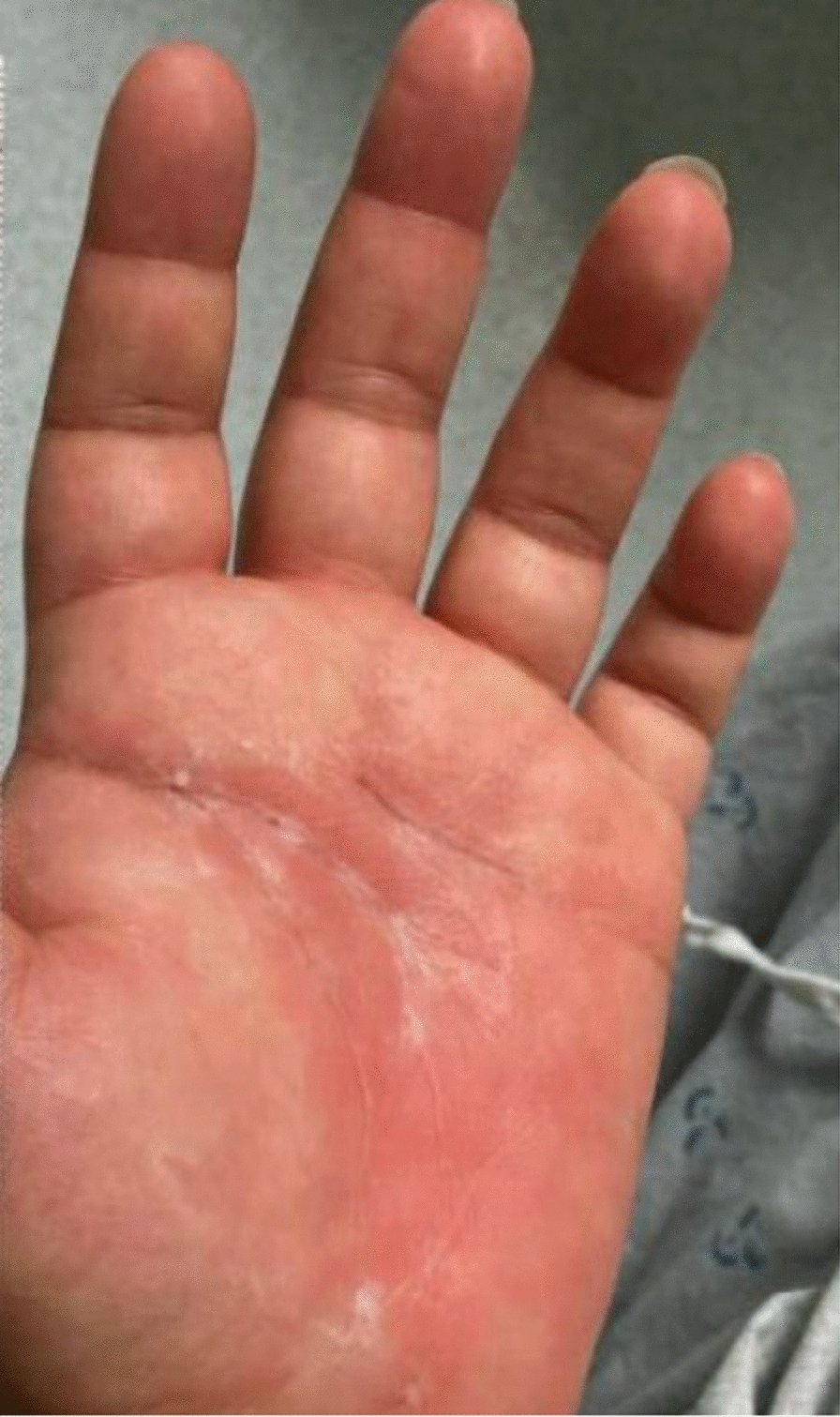
Erythematous rituximab-induced rash on palmar aspect of left hand

**Fig. 3:**
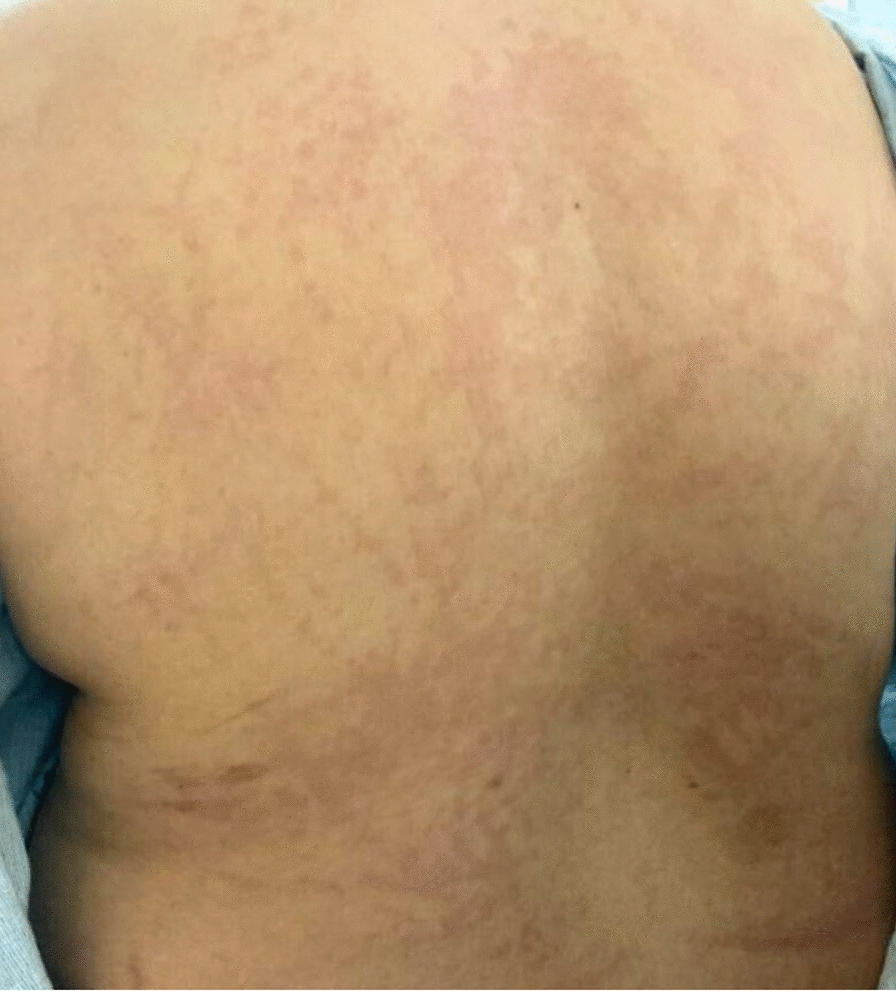
Rituximab-induced rash with scattered erythema and superimposed excoriations

A 34-year-old Mexican–American female patient with focal segmental glomerulosclerosis, managed with cyclosporine and low-dose prednisone, and chronic cervical and submandibular lymphadenopathy, presented to the hematology-oncology clinic with a 5-year history of persistent neck swelling. She denied systemic symptoms, such as weight loss or night sweats, however, her lymphadenopathy persisted. An excisional lymph node biopsy performed 2 months prior revealed focal follicle lysis and increased IgG4 expression, leading to a diagnosis of PTGC. Due to symptomatic lymphadenopathy, treatment with rituximab was initiated [5]. Although the first two infusions were well tolerated, she presented 1 day after her third infusion with bilateral upper extremity, chest, and back pain, as well as a pruritic, erythematous, macular rash on her face (Figs. 1, 2). Initial labs revealed elevated inflammatory markers [erythrocyte sedimentation rate (ESR) 52 mm/hour, C-reactive protein (CRP) 5.25 mg/L], mild anemia, hypoalbuminemia (1.6 g/dL), normal complement levels, and no leukocytosis. Urinalysis demonstrated moderate proteinuria with mild hematuria, but no renal imaging studies were considered necessary. The next day, the patient endorsed new-onset bilateral hand pain and numbness with an associated erythematous, tender rash on the left palm, indicating worsening of her underlying condition (Fig. 3). The patient was started on a prednisone taper and as-needed diphenhydramine for serum sickness secondary to rituximab treatment. At clinic follow-up 1 week later, the patient reported complete resolution of her symptoms, and future rituximab infusions were canceled to prevent recurrence. At the patient’s 9-month follow-up, the IgG4-related lymphadenopathy was stable without any further treatment.

## Discussion and conclusions

Rituximab-induced serum sickness (RISS) was first documented in 2002 when patients treated for autoimmune polyneuropathy and immune thrombocytopenic purpura (ITP) experienced fever, malaise, and polyarthralgia within 6–10 days post-treatment [6]. Since then, RISS has been primarily associated with management of autoimmune diseases. This case underscores the importance of prompt recognition and management of rituximab-associated adverse reactions in rheumatologic, hematologic, and inflammatory conditions such as IgG4-RD to prevent symptom progression and minimize complications [7].

This case also highlights an unusual presentation of IgG4-RD, as PTGC is typically seen in older male patients. While IgG4-RD presents with a range of organ-specific symptoms, PTGC is often characterized by asymptomatic lymphadenopathy [8, 9]. Treatment for IgG4-RD is indicated in cases of distal disease spread, such as pancreatic or renal involvement, or biochemical abnormalities, such as elevated transaminases or creatinine, which signify disease progression without overt symptomatology. Glucocorticoids are often the first-line treatment, however, their efficacy in long-term disease management is limited, often failing to achieve sustained disease control [5]. The standard initial treatment regimen for IgG4-RD consists of high-dose glucocorticoid monotherapy, followed by a gradual taper over several weeks until symptom resolution [10, 11]. For those with contraindications to steroids, steroid-sparing agents such as rituximab, azathioprine, or mycophenolate are viable options. Rituximab, however, is commonly preferred due to its superior efficacy [5]. Additionally, rituximab has demonstrated greater effectiveness in younger patients and those with longstanding symptomatic disease, making it a primary treatment consideration for our patient [12]. Before starting rituximab infusions, it is crucial to assess risk factors for RISS, including high drug dosage, advanced age, and hypergammaglobulinemia. As this case demonstrates, RISS can occur not only in rheumatologic and hematologic disease, but also in immune-mediated fibroinflammatory diseases such as PTGC IgG4-RD. In otherwise asymptomatic and localized IgG4-RD, biannual follow-ups and laboratory monitoring are typically sufficient to adequately monitor disease, thereby reducing medication use and associated risks.

This case is notable for the rapid onset of symptoms 1 day after rituximab treatment despite the absence of hypergammaglobulinemia or complement deficiency, which are known risk factors for RISS [13]. Additionally, this is the first documented case of RISS in IgG4-RD and emphasizes the importance of close patient monitoring following rituximab therapy to promptly identify complications. If RISS occurs, immediate treatment cessation is recommended to prevent further attacks, however, select studies suggest that re-treatment with rituximab can be cautiously considered with pre-treatment glucocorticoids [14].

## Data Availability

Not applicable.

## Abbreviations

RISS: Rituximab-induced serum sickness
FSGS: Focal segmental glomerulosclerosis
IgG4-RD: Immunoglobulin G4-related disease
PTGC: Progressive transformation of germinal centers
